# Sink-index: a network-based EEG marker for frontotemporal dementia and Alzheimer’s disease

**DOI:** 10.1093/braincomms/fcaf259

**Published:** 2025-06-30

**Authors:** Luis A Sanchez, Surya Pandiaraju, Autumn O Williams, Amir H Daraie, Chiadi U Onyike, Sridevi V Sarma

**Affiliations:** Department of Biomedical Engineering, Johns Hopkins University School of Medicine, Baltimore, MD 21205, USA; Department of Biomedical Engineering, Johns Hopkins University School of Medicine, Baltimore, MD 21205, USA; Department of Biomedical Engineering, Johns Hopkins University School of Medicine, Baltimore, MD 21205, USA; Department of Biomedical Engineering, Johns Hopkins University School of Medicine, Baltimore, MD 21205, USA; Department of Psychiatry and Behavioral Sciences, Johns Hopkins University School of Medicine, Baltimore, MD 21205, USA; Department of Biomedical Engineering, Johns Hopkins University School of Medicine, Baltimore, MD 21205, USA

**Keywords:** sink-Index, dynamical systems, EEG marker, Alzheimer, frontotemporal dementia

## Abstract

Frontotemporal dementia is a complex neurodegenerative illness characterized by a progressive deterioration in temperament, judgement, behaviour, and communication. Misdiagnosis and late diagnosis occur frequently due to the complexity of the phenotypes, overlaps of features with other neurodegenerative syndromes and psychiatric disorders, and ill-defined preclinical phases of the illness. Diagnosis relies on structural or functional brain imaging to show characteristic atrophy, hypoperfusion or hypometabolism profiles. The sensitivity of neuroimaging is lower in the earliest phases of the illness, and there are few alternatives. Scalp electroencephalography (EEG) is a widely available, low-cost technology, but its utility in the differential diagnosis of dementia will require EEG indices of high sensitivity and discriminatory value. We have used scalp EEG to develop subject-specific Dynamic Network Models, from which we summarize the reciprocal relationships between the nodes (defined by the EEG channel). This index, the ‘Sink-Index’, characterizes how activity in each node (or channel) responds to activity in other nodes in the network. In this context, ‘sources’ are nodes that exert significant influence on the activity of different regions but are not themselves influenced, whereas ‘sinks’ represent influenced regions that do not affect activity in others. We hypothesized that brain regions associated with Frontotemporal dementia and Alzheimer's disease syndromes behave as sinks and have higher sink indices than healthy brain regions. This hypothesis was tested in a cohort of 88 subjects: 23 with frontotemporal dementia, 36 with Alzheimer's disease, and 29 healthy controls. The Sink-Index of nodes in the frontal–temporal and central–parietal–occipital brain regions differed between Frontotemporal dementia (1.3389 ± 0.0895 versus 0.8444 ± 0.0651), Alzheimer’s disease (0.6015 ± 0.0188 versus 0.7766 ± 0.0158), and healthy controls (0.8978 ± 0.0453 versus 0.9116 ± 0.0457). These findings suggest the Sink-Index is an EEG marker with utility for the differential diagnosis of dementia syndromes.

## Teaser

Sanchez *et al*. report that the novel Sink-Index, an EEG marker derived from dynamic network models, distinguishes Alzheimer’s disease and Frontotemporal Dementia from Healthy Controls with high accuracy. The study highlights the Sink-Index’s potential as a cost-effective, non-invasive diagnostic tool, enhancing differential diagnosis and understanding of neurodegenerative network pathophysiology.

## Graphical Abstract

**Graphical Abstract:**
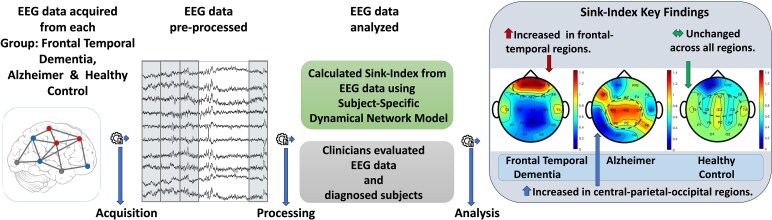

## Introduction

Frontotemporal dementia (FTD) is a clinically complex neurodegenerative illness characterized by progressive deteriorations in temperament, judgment, conduct, and communication.^1^ FTD is, along with Alzheimer’s disease (AD), a leading cause of dementia in adults younger than 60 years of age.^2^ Diagnosis requires careful clinical interview and examination to identify the syndrome by describing the principal characteristics, chronology, and tempo, and radiological profiles that provide crucial health support.^3^ Misdiagnosis and late diagnosis are not uncommon, owing to the complexity of the phenotypes and their overlap with those of other neurodegenerative syndromes and psychiatric disorders.^4^ Furthermore, the preclinical features have not yet been established—confounding diagnosis in those cases in which the features have not yet developed into a typical syndrome.^5,6^

It is standard diagnostic practice to complement clinical examinations with brain imaging tools such as MRI,^7^ single photon emission computed tomography (SPECT),^8^ and fluorodeoxyglucose positron emission tomography (FDG-PET).^9^ These methods capture the patterns of neurodegeneration characteristic of each syndrome–focal atrophy or hypometabolism of the frontal and/or anterior temporal regions in FTD, and diffuse atrophy or hypometabolism with predilection for the hippocampi, medial temporal lobe, and the temporoparietal cortex in AD.^10^  Ad diagnosis can be confirmed using CSF assays of amyloid and tau, or amyloid PET imaging,^11^ but these technologies are not yet in wide use. Serum neurofilament light chain levels predict which carriers of causal genetic mutations will develop an FTD prodrome.^12^ However, Neurofilament Light Chain (NfL) levels vary depending on genetic and clinical factors,^12^ and NfL elevation occurs in other neurodegenerative diseases—it is not specific to FTD.

In resource-poor environments, MRI, SPECT, and PET are often inaccessible, owing to their costs.^13,14^ Thus, there would be much value in a diagnostic test that detects dementia-related physiological changes in the early stages, distinguishes FTD from AD and other neurodegenerative conditions, and is widely accessible. Scalp electroencephalography (EEG) is an established, widely available, non-invasive, low-cost technology that provides an intriguing alternative; however, utility for early diagnosis requires EEG markers that discriminate between dementia syndromes.^15^

While normal EEG tracings are considered EEG markers for healthy controls, researchers have yet to identify reliable EEG markers for AD and FTD.^15^ However, strides have been made in utilizing EEG data to study AD and FTD through various signal analysis techniques. For example, studies employing time-domain analyses have documented a decrease in signal complexity in EEG readings of AD subjects by using Approximate Entropy (ApEn)^16,17^ and Sample Entropy (SampEn).^18,19^ Other time-domain analyses have used connectivity features and support vector machine (SVM) algorithms to classify dementia subjects (AD + FTD) from HC, or AD subjects from FTD, with 73% accuracy.^20^ Frequency domain studies employing Power Spectrum analysis in EEG rhythms and classification algorithms to distinguish AD, FTD, or HC subjects have achieved 85–93% accuracy rates.^21,22^ However, these studies use small datasets (less than 40 subjects total) and usually examine distinctions between dementia cases and healthy subjects; they do not address the differential diagnosis problem. Similarly, several studies using Discrete Fourier Transform and Wavelets to analyse signals in both time and frequency to provide a comprehensive view of how frequencies vary over time, in combination with algorithms for classifying subjects as AD, FTD, or HC, have reported accuracy rates ranging from 83 to 86%.^23,24^ As in the frequency domain studies, these time-frequency domain studies utilize small samples or do not address FTD versus AD classification. Furthermore, the aforementioned techniques use individual EEG channel features or pairwise and static connectivity features, which cannot capture the n-to-n regional dynamics across the brain network. In other words, despite these advancements, research is yet to deliver an EEG marker that reliably detects AD and FTD and distinguishes one from the other.

In this study, we used EEG recordings to construct subject-specific Dynamic Network Models (DNM) to derive a computational EEG marker for dementia detection. These DNMs capture brain regions’ spatial interactions and temporal influences on the brain network, thereby identifying pathological network nodes (defined by EEG channels). We observed that certain pathological network nodes had diminished influence on the network and increased susceptibility to influence by other nodes in the network, the latter suggesting compensatory mechanisms. Specifically, we employed a novel scalp EEG marker called the Sink-Index that captures the dynamics of n-to-n nodal influence in a subject's brain network, quantifying connectivity properties and the interplay between various brain regions.

Studying both Alzheimer's Disease (AD) and Frontotemporal Dementia (FTD) in a comparative framework offers several key insights. Although AD and FTD are distinct neurodegenerative disorders with differing clinical and radiological profiles,^25^ differential diagnosis can be challenging, especially in their early stages. Misdiagnosis between these conditions is common due to overlapping symptoms such as memory and language impairments, and behavioural dysfunctions, particularly when there are atypical presentations.^26^ By analysing the performance of the EEG-derived Sink-Index measures in FTD and AD cohorts, we are able to examine their relative utility for case detection as well as their potential for differential diagnosis. This comparative approach is essential for developing a robust EEG marker that not only distinguishes these two disorders but also enhances diagnostic precision across dementia syndromes. In the future, comparative studies may offer opportunities to explore the utility of the Sink-Index for examining the mechanisms of network dysfunctions.

Utilizing a publicly available scalp EEG dataset,^27^ which includes recordings from 88 Ad, FTD, and HC subjects, we applied our analysis pipeline, which involved creating a subject-specific DNM to calculate the Sink-Index across all brain regions. We analysed the Sink-Index from the combined Frontal–Temporal (FT) versus Central–Parietal–Occipital (CPO) regions and incorporated the ratio of these measurements as a feature into several supervised learning algorithms. Accuracy results, measured in terms of the Area Under the Curve (AUC) and precision, obtained from a leave-one-out cross-validation method, suggest that subject-specific DNMs combined with the Sink-Index outperform traditional EEG spectral and time-frequency analysis. These findings suggest an accurate and cost-effective alternative for capturing brain dysfunction for the differential diagnosis of dementia.

## Materials and methods

### Dataset description

We analysed data from the OpenNeuro website containing EEG recordings from AD, FTD and HC subjects.^27^ These subjects had undergone evaluation and diagnosis at the 2nd Department of Neurology at AHEPA General Hospital in Thessaloniki. Diagnosis of Frontotemporal Dementia (FTD) and that of Alzheimer's disease (AD) in this study were based on standard clinical guidelines, i.e. the Rascovsky Criteria for FTD,^28^ and the McKhann criteria for AD.^29^ Neuroimaging results were used to support clinical classifications, as specified in the criteria. There were 88 subjects; 36 with AD, 23 with FTD, and 29 HC. Notably, none of the subjects reported any comorbidities. Table 1 summarizes the population statistics.^30^ The Mini-Mental State Examination (MMSE) scores varied significantly among the groups. The AD group had an average score of 17.75 (±4.5), indicating a moderate level of cognitive impairment. The HC group presented with an average score of 30 (±0), reflecting normal cognitive function. In contrast, the FTD group showed an average MMSE score of 22.1 (±2.6), suggesting mild cognitive impairment.^30^

**Table 1:** Dataset demographics

	Diagnosis		
Features	FTD	Alzheimer	Healthy
Number of patients	23	36	29
Gender, male/female	14/9	12/24	18/11
Age, years	63.7 (±8.22)	66.4 (±7.88)	67.8 (±5.4)
MMSE	22.1 (±2.64)	17.75 (±4.5)	30
EEG Recording, minutes	12 (±4.5)	13.5 (±8.1)	13.8 (±2.0)

Dataset statistical analysis (values in parenthesis represent standard deviation).

### Data acquisition and pre-processing

This study analysed resting-state EEG data. For creating the recordings, the clinical team at the AHEPA General Hospital used the Nihon Kohden EEG 2100 device configured in a standard 10–20 montage and operated at a sampling rate of 500 Hz, with a resolution of 10μV/mm. During the recording sessions, participants were seated in a relaxed posture with their eyes closed to minimize external visual stimuli and facilitate resting-state brain activity. No cognitive tasks were performed during the recordings, ensuring the data represent spontaneous neural activity at rest. These conditions were standardized across all participants to maintain consistency in data collection.^30^

We down-sampled all recordings to 250 Hz, filtered them using a Butterworth band-pass filter (BPF) 0.5–48 Hz, and notch filtered them at 50 Hz and their harmonics with a stopband of 2 Hz to remove power line noise interference. For high-amplitude EEG artefact correction, we used the Artefact Subspace Reconstruction routine (ASR) included in the MATLAB plugin EEGLab version 2023.1.^31^ We further performed automatic eye and jaw artefacts removal using the independent component analysis algorithms (RunICA, ICLabel) in EEGLab.^32^ We used MATLAB R2023b Update 7 (MathWorks, 2024) to process and analyse the data. We built models, Receiver Operating Curves (ROCs), AUCs, and Confusion Matrices using Python 3.12.0 (Python Software Foundation, Wilmington, DE).

### Dynamic network models

Dynamic Network Models are generative models that describe the evolution of complex systems composed of interconnected components. These models are particularly useful for characterizing how network structures change over time in response to internal and external factors. In the context of EEG signals, DNMs reveal how each channel dynamically interacts with and influences the rest of the brain network, offering insights into the system's connectivity, stability, controllability, and other internal properties such as bandwidth and system gain.^33^

For this study, we constructed Linear Time-Varying (LTV) DNMs for each subject based on their EEG recordings. Each DNM was formed by concatenating a sequence of discrete-time Linear Time-Invariant (LTI) models, calculated over specified time windows ***T***. For each subject, the model takes the form:


(1) x(t+1)=Ax(t)+e(t)

where $x(t)\;\in \;R^{Nx1}$ is the EEG ‘state’ vector representing the brain's dynamic state at time *t*, $A\;\in \;R^{NxN}$ is the state transition matrix (distinct from an adjacency matrix in graph theory), which captures the interactions between EEG channels over time, and $e(t)\in R^{N\times 1}$ is a white Gaussian noise vector independent of the state x(t), and ***N*** denotes the number of EEG channels.

The state transition matrix A was estimated for each 125 ms time window using a least squares method,^34^ resulting in a sequence of A matrices that collectively form the linear time-varying DNM for the recording. The sum of all ***T*** windows corresponds to the total duration of the recording. The resulting DNMs allow for the analysis of system properties such as fragility and connectivity, as well as the identification of patterns in brain activity under different conditions. By employing this approach, we accurately captured the dynamics of EEG channel interactions, providing deeper insights into the brain's network behaviour.

### Sink-Index computation from DNM

In 2022, Gunnarsdottir *et al*. used intracranial EEG (iEEG) data to create subject-specific LTV DNMs using Eq. (1) from iEEG electrode signals.^35^ The authors conceptualized iEEG channels or iEEG nodes as ‘sources’ and ‘sinks’ in the brain network. In their approach, ‘sources’ denote brain regions that exert significant influence over other nodes without being influenced by the network. Conversely, ‘sinks’ are regions predominantly influenced by other nodes without exerting much influence themselves.

### Computing source-sink indices from DNMs

**Figure 1:**
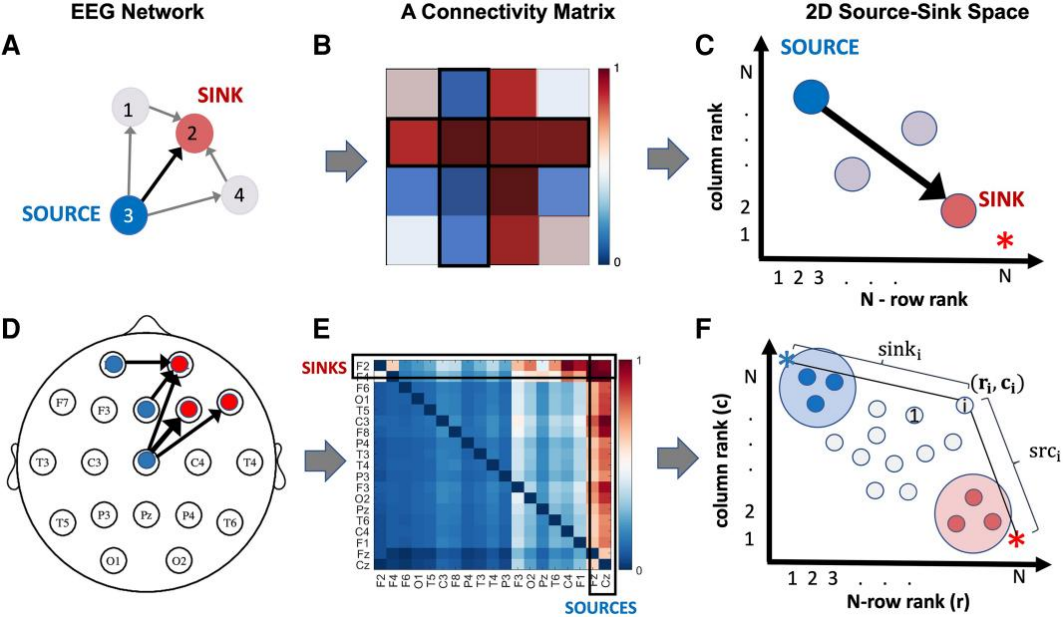
**Computing source-sink indices from DNM.** (**A**) 4-node network example. (**B**) Corresponding A matrix. (**C**) 2D source-sink representation of network. (**D**) N-node scalp EEG network example. (**E**) Corresponding A matrix. (**F**) 2D source-sink representation of the network with sink index (sink_i_) and source index (src_i_), labelled (defined in equations 1 and 2, respectively).

The DNM, i.e. the sequence of matrices $\{A_{1},\;A_{2},\ldots ,A_{T}$ }, will be used to identify two special groups of nodes in the resting-state-scalp EEG network in each ***T*** = 125 msec window: those that are continuously inhibiting a set of their neighbouring nodes (denoted as ‘sources’) and the inhibited nodes themselves (denoted as ‘sinks’). Consider the 4-node network example in Fig. 1. Node 3 is a source node because it is highly influential on all nodes in the network and is not being influenced itself. In contrast, node 2 is a sink node because it is highly influenced by other nodes, specifically node 3, and does not influence any other node in the network. This is reflected in the rows and columns of the ***A*** matrix of the DNM, respectively. A sink node will have nearly all zeros in its column vector in the ***A*** matrix (blue in Fig. 1B) because it does not impact the future activity of its neighbours; but its row vector will contain large values (closer to 1 in Fig. 1B) because it is highly influenced by all the other nodes. Source nodes have the opposite properties in the ***A*** matrix. Their columns have larger values (closer to 1) while the rows have smaller values (closer to 0). See column and row 3 of source node 3 in Fig. 1B. Note that diagonal entries are typically nonzero as they reflect self-loops in the population of neurones. All nodes can be quantified as more of a sink or more of a source by computing the 2-norm of the columns (sum of squared elements) and rows of the ***A*** matrix and ranking them against each other (where rank 1 indicates smallest norm, rank *N* is largest norm for network with N nodes). When drawn in the 2D space shown in Fig. 1C, sources are located at the top left (high column rank and low row rank), while sinks are located at the bottom right (low column rank and high row rank). Figure 1D-F illustrates source-sink analysis from a scalp EEG network with more nodes. In our subject data sets, the scalp EEG consists of 19 channels.

We then compute the Sink-Index for each EEG channel from the 2D source-sink representation of the network shown in Fig. 1F derived for each ***A*** matrix in the DNM. The Sink-Index captures how close a node is to the ideal sink, which is a node whose row rank is N (number of channels) and whose column rank is 1 (see Fig. 1F red star). The Sink-Index is computed as the following 2-norm (Euclidean distance):


(2) $$\;\;\sin k_{i}=\sqrt{2}-\|(rr_{i},cr_{i})-\left(1,\frac{1}{N}\right)\|\;\mathrm{for}\;i=1,2,\ldots ,N$$

where $rr_{i}=\sqrt{\sum_{\;j=1}^{N}{\left(A_{ij\;}\right)}^{2\;}}$ is the ‘size’ of the *i*th row and $cr_{j}=\sqrt{\sum_{i=1}^{N}{\left(A_{ij\;}\right)}^{2\;}}\;$ is the ‘size’ of the jth column of ***A***. The larger this index, the more likely a sink. Specifically, the two norms were computed and then ranked and normalized such that the largest *rr* and *cr* value was 1, and the smallest value was 1/N.

Based on the source-sink theory, generating subject-specific DNM and calculating the Sink-Index across all EEG channels could hypothetically show that frontal and temporal brain regions in FTD act as strong sinks. This assumption leads us to hypothesize that the Sink-Index distribution might be more heterogeneous in AD, potentially enabling differentiation from HCs, who would not exhibit pronounced sources or sinks. Further empirical research is needed to validate these hypotheses and determine their accuracy and value in clinical settings.

### Analysis pipeline

**Figure 2:**
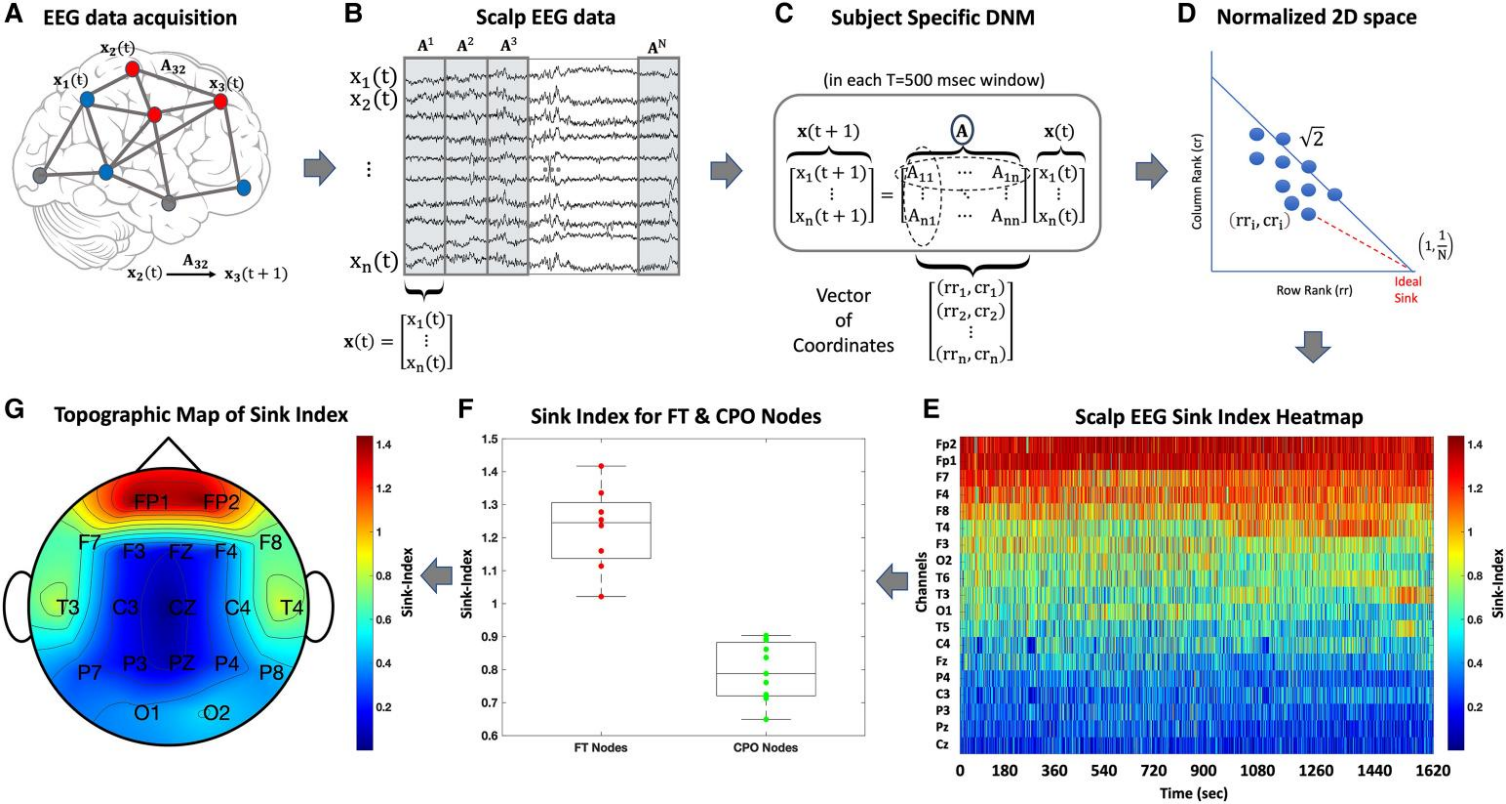
**Sink-Index analysis pipeline.** The Sink-Index (SI) is a unitless measure that visualizes the inter-dynamics of brain networks, highlighting regions with impaired function. (**A**) Scalp EEG recordings from individual subjects serve as the foundation for Sink-Index (SI) computation. (**B**) The recordings are segmented into non-overlapping time windows of duration T. (**C**) For each segment, a state evolution matrix is estimated using least squares. (**D**) A coordinate system is constructed from row and column rank sums per channel, forming rank coordinate pairs ($rr_{i}$, $cr_{i}$). (**E**) These coordinates are used to compute the Sink-Index for each channel, visualized as a heatmap where higher values indicate stronger sink dynamics. (**F**) The SI is averaged within the frontal–temporal (FT) and central–parietal–occipital (CPO) channel groups and compared across subjects and cohorts using box plots. Each individual data point in the boxplot represents the average Sink-Index for a single subject within the specified node group. In all subjects, the frontal–temporal regions included *N* = 8 nodes (Fp1, Fp2, F3, F4, F7, F8, T3, and T4), while the central–parietal–occipital regions included *N* = 11 nodes (O1, T5, O2, T6, Cz, Pz, P3, Fz, P4, C3, and C4). (**G**) Brain topographic maps illustrate the spatial distribution of the Sink-Index, highlighting regions with altered functional dynamics.

Figure 2 depicts the analysis pipeline used for the Sink-Index portion of this study. For each subject scalp EEG recording (Fig. 2A), we separated the recording into smaller intervals of time duration ***T*** (Fig. 2B). We calculated the state evolution matrix for each interval *T* using least square estimation (Fig. 2C). For each matrix *k*, we compute all row and column rank coordinate pairs $(rr_{i}\;,\;cr_{i})\;$ for each channel as described above and shown in Fig. 2D. We then substituted the coordinates for each channel into Eq. (2) and found the Sink-Index for channel *i* of matrix *k*. We repeated the process for N channels and M matrices, resulting in a Sink-Index vector of size N per time, which we plotted into a heatmap where the heat is the Sink-Index as calculated in Eq. 2 (Fig. 2E). We grouped the channels into two groups, namely, the frontal–temporal, FT, group (Fp1, Fp2, F3, F4, F7, F8, T3, and T4) and the central–parietal, occipital, CPO, group (O1, T5, O2, T6, Cz, Pz, P3, Fz, P4, C3, and C4). For each group of channels, we calculated the average Sink-Index across the entire group and box plotted both FT and CPO groups per subject, per cohort, across cohorts, and as a Sink-Index FT/CPO ratio across all subject groups (Fig. 2F). Finally, we displayed the Sink-Index as a brain topographic map, as shown in Fig. 2G.

### Spectral and time-frequency analysis

We perform the Sink-Index analysis in the time domain from subject-specific DNMs. We also analysed the data using frequency and time-frequency techniques for comparison and validation. In the frequency domain, we performed an average frequency power analysis. We decomposed the signal into functionally distinct frequencies at (1–4 Hz), (4–8 Hz), (8–12 Hz), and (12–30 Hz) corresponding to the delta, theta, alpha, and beta bands. We calculated the Fast-Fourier transform for each band and squared the magnitude to obtain the power spectrum. We averaged the power spectrum across the entire recording for frontal–temporal and central–parietal groups and box plotted both FT and CPO groups per subject and cohort across cohorts.

The time-frequency analysis used wavelet decomposition and Hjorth parameters to extract samples by taking eight-second windows with 50% successive overlap. Five-level dyadic wavelet decomposition (using ‘sym4’, ‘coif2’, ‘haar,’ and ‘db4’ templates) helped capture the data's temporal and spectral features across all channels. At the same time, Hjorth parameters (activity, mobility, and complexity) provided complementary measures of the neural activity. Principal Components Analysis (PCA) provided feature reduction from an initial 285 features averaged across all 8-second windows to five features per subject.

### Feature extraction and classification

Supervised learning algorithms provide a mechanism to predict and infer relationships between data features and targets. We contrasted the accuracy, specificity, and sensitivity of seven different algorithms, namely linear discriminant analysis, quadratic discriminant, k-nearest neighbour, naïve Bayes, decision trees, random forest, logistic regression, and SVM to select the best classifier for AD, FTD, and HC. The Sink-Index ratio, frequency band energy, and Hjorth parameters provided the features of the classification algorithms for each of the signal analysis techniques used in the time, frequency, and time-frequency domains. The classification problem addressed was multiclass using the one-versus-the-rest approach, which compares the positive class against the combination of the remaining classes, considered as aggregate, the negative class, and assumed to be one. This approach tested the ability of the features to classify in the presence of all conditions correctly. We used three distinct features for classification based on the signal analysis technique. We used the ratio derived from the averaged FT nodes Sink-Index divided by the averaged combined CPO nodes Sink-Index for the Sink-Index analysis. For the frequency analysis, the power spectrum across the entire recording for frontal–temporal and central–parietal–occipital groups served as features, while in time-frequency analysis, the PCA-reduced Hjorth parameters and wavelet coefficients provided the corresponding classifying feature needed by the algorithms.

### Statistical analysis

To compare the Sink-Index medians between different brain regions (FT and CPO), we utilized the Mann–Whitney (MW) U-test, which is well-suited for non-normally distributed data. The values for the U-test statistic indicate the rank sums’ relative differences, with smaller U-values corresponding to more significant *P*-values. To control for multiple comparisons, a Bonferroni correction was applied, setting stricter thresholds for statistical significance (e.g. *P* < 0.01).

To evaluate differences across groups (FTD, AD, and HC), we applied the Kruskal–Wallis (KW) test, a non-parametric method that identifies significant differences in distributions across multiple groups. The Kruskal–Wallis test statistic (*H*) measures the extent to which the rank distributions differ among groups, with higher *H* values indicating greater differences and corresponding to smaller *P*-values based on a chi-square distribution, where *k* is the number of groups being compared and the degrees of freedom is *k*-1. *Post hoc* pairwise comparisons were conducted using Dunn’s test with Bonferroni correction to minimize Type I error. The Dunn test statistic is a *z*-score that quantifies the difference in mean ranks between two groups, with larger absolute *z*-scores indicating greater differences and corresponding to smaller *P*-values. These comparisons helped to pinpoint specific inter-group differences. In this study, statistical significance was categorized as follows: (i) Highly significant (*P* < 0.001): Indicated with ‘***’; (ii) Very significant (*P* < 0.01): Indicated with ‘**’; (iii) Significant (*P* ≤ 0.05): Indicated with ‘*’; (iv) Not significant (*P* > 0.05): Denoted as ‘n.s.’.

Given the relatively small dataset of fewer than 100 subjects, we implemented a leave-one-out cross-validation (LOOCV) procedure to optimize reliability, limit bias, and maximize accuracy in the performance estimates of our models. This approach involved splitting the data into two subsets: a training set containing all but one subject (87 subjects) and a test set containing the excluded subject. This process was repeated 88 times, ensuring that every subject was used as the test set exactly once. The hyperparameter k was set to two to structure these subsets effectively.

Model performance was assessed using Receiver Operating Characteristic (ROC) metrics, which evaluate the quality of multiclass classifiers. True positive rates (TPR) and false positive rates (FPR) were plotted, and the contributions of all classes were micro-averaged. Specifically, TPR and FPR were calculated using equations (3) and (4), respectively:


(3) $$TPR=\;\frac{\sum_{i=1}^{C}\;TP_{i}}{\sum_{i=1}^{C}\;(TP_{i}+\;FN_{i})}$$

(4) $$FPR=\;\frac{\sum_{\;j=1}^{C}\;FP_{j}}{\sum_{\;j=1}^{C}\;(FP_{j}+\;TN_{j})}$$

where C denotes the total number of classes, TP the true positives, FP the false positives, FN the false negatives, and TN the true negatives. These metrics provided a foundation for computing the AUC, which served as a measure of model performance. Additionally, accuracy, precision, sensitivity, and specificity were derived to compare the performance of various analysis techniques using AUC and precision as benchmarks. We also assessed the relationship of Sink-Index ratios with variables such as age, sex, and MMSE scores. Statistical significance for these relationships was evaluated using the same significance thresholds described above.

## Results

### The sink-Index highlights regions of brain pathology in the FTD and AD groups

**Figure 3:**
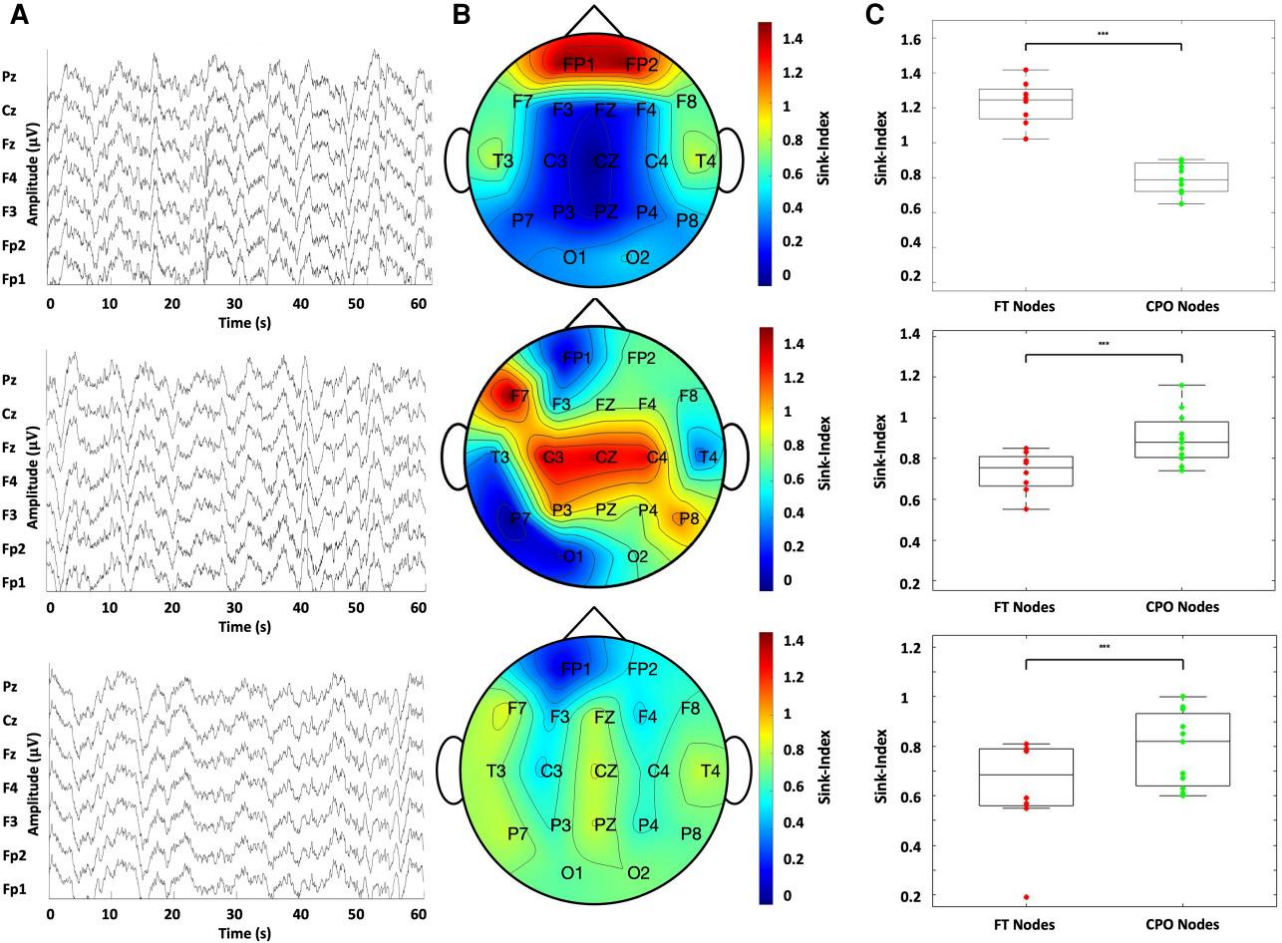
**Three subjects Sink-Index examples.** Subject 1 (top) is an FTD patient. Subject 2 (middle) is an AD patient, and Subject 3 (bottom) is an HC subject. (**A**) After pre-processing, we displayed only selected channels and showed each subject ten to 30 min of scalp EEG data. (**B**) Brain Topographic Maps of each subject using Sink-Index vectors across all matrices for each subject’s recording. (**C**) Box plots of the average Sink-Index (across the entire recording) for the frontal–temporal and the central–parietal–occipital regions. Pathological brain regions for each condition present higher sink indices. For all three subjects, the Mann–Whitney U-test revealed statistically significant differences between the average sink indices of the FT and CPO nodes. The *P*-values were *P* = 0.0000, *P* = 0.0006, and *P* = 0.0053 for Subjects 1, 2, and 3, respectively. For each subject, the frontal–temporal regions included *N* = 8 nodes (Fp1, Fp2, F3, F4, F7, F8, T3, and T4), while the central–parietal–occipital regions included *N* = 11 nodes (O1, T5, O2, T6, Cz, Pz, P3, Fz, P4, C3, and C4).

Averaging the Sink-Index over the entire recording gave us one data point per channel per subject. Figure 3 illustrates the scalp EEG, brain topographic map, and average Sink-Index across all channels grouped by FT and CPO regions for three subjects: FTD (Subject 1), AD (Subject 2), and HC (Subject 3). Bonferroni corrected *P*-values from Mann–Whitney U-tests provided a robust statistical comparison of the sink-index medians between FT and CPO nodes across the three different subjects, ensuring a stricter significance threshold to control for Type I errors. Subject 1 is a 57-year-old female with an MMSE score of 22. Here, the FT nodes display highly significant (*P* = 0.000, U = 0) sink indices than the CPO nodes, corresponding to the standard atrophy (or hypometabolism) profile of FTD. The brain topographic map shows higher sink indices in the Fp1 and Fp2 nodes corresponding to the prefrontal regions. Likewise, T3 and T4 nodes in the FT region present higher sink indices than those in the CPO region. The boxplot confirms the brain topographic map results.

AD Subject 2 is a 70-year-old male with an MMSE score of 14. In this case, the FT nodes display highly significant (*P* = 0.0006, U = 1) sink indices than the CPO nodes corresponding to the radiological profile for AD. The topographic map of the brain shows higher sink indices in the F7, C3, Cz, C4, and P8 nodes corresponding to the frontal, central, and parietal regions. The boxplot confirms higher sink indices in the CPO regions. HC Subject 3 is a 67-year-old male with an MMSE score of 30. This case presents balanced, very significant (*P* = 0.0053, U = 7) average sink indices for FT and CPO regions. This subject displays relatively low sink indices in topographic brain maps and similar numbers in the boxplot of the subject’s brain.

### Common cohort-level characteristics emerge within FTD, AD, and HC

**Figure 4:**
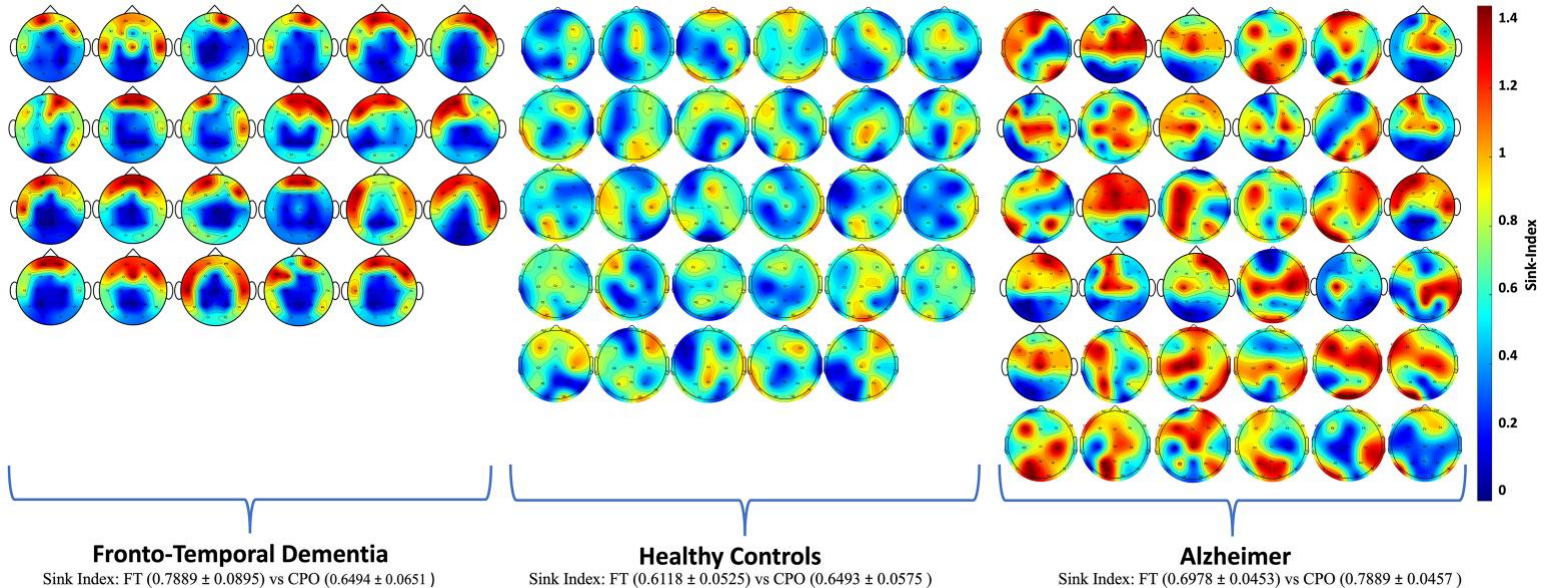
**Brain topographic maps per cohort.** These topographic maps illustrate average SI values across regions of interest for FTD, HC, and AD cohorts (from left to right). There were 88 subjects in total: AD (*N* = 36), FTD (*N* = 23), and HC (*N* = 29). FTD subjects exhibit elevated SI values predominantly in frontal–temporal (FT) regions, while AD subjects show higher SI values in central–parietal–occipital (CPO) regions. These patterns align with radiological findings of focal atrophy in FTD and diffuse atrophy in AD. Healthy controls (HC) demonstrate balanced SI distributions, indicating preserved network dynamics. The heatmap intensity reflects the magnitude of the SI: brighter regions indicate greater decoupling, representing reduced influence from other regions, while darker regions indicate stronger coupling, representing increased influence from other regions. The Sink-Index (SI) is a unitless measure that ranges from 0.00 to 1.44.

Figure 4 depicts the topographic maps of the brains for each cohort. The topographic maps and sink indices reveal that FTD and HC groups exhibit more homogeneous patterns, with FTD subjects showing concentrated activity in the FT regions and HCs displaying a balanced distribution of Sink-Index magnitudes across the brain. In contrast, the AD cohort demonstrates a more heterogeneous pattern, with higher sink indices in the CPO regions. Some AD subjects also exhibit high indices in the FT regions, corresponding to the expected radiological profile.

**Figure 5:**
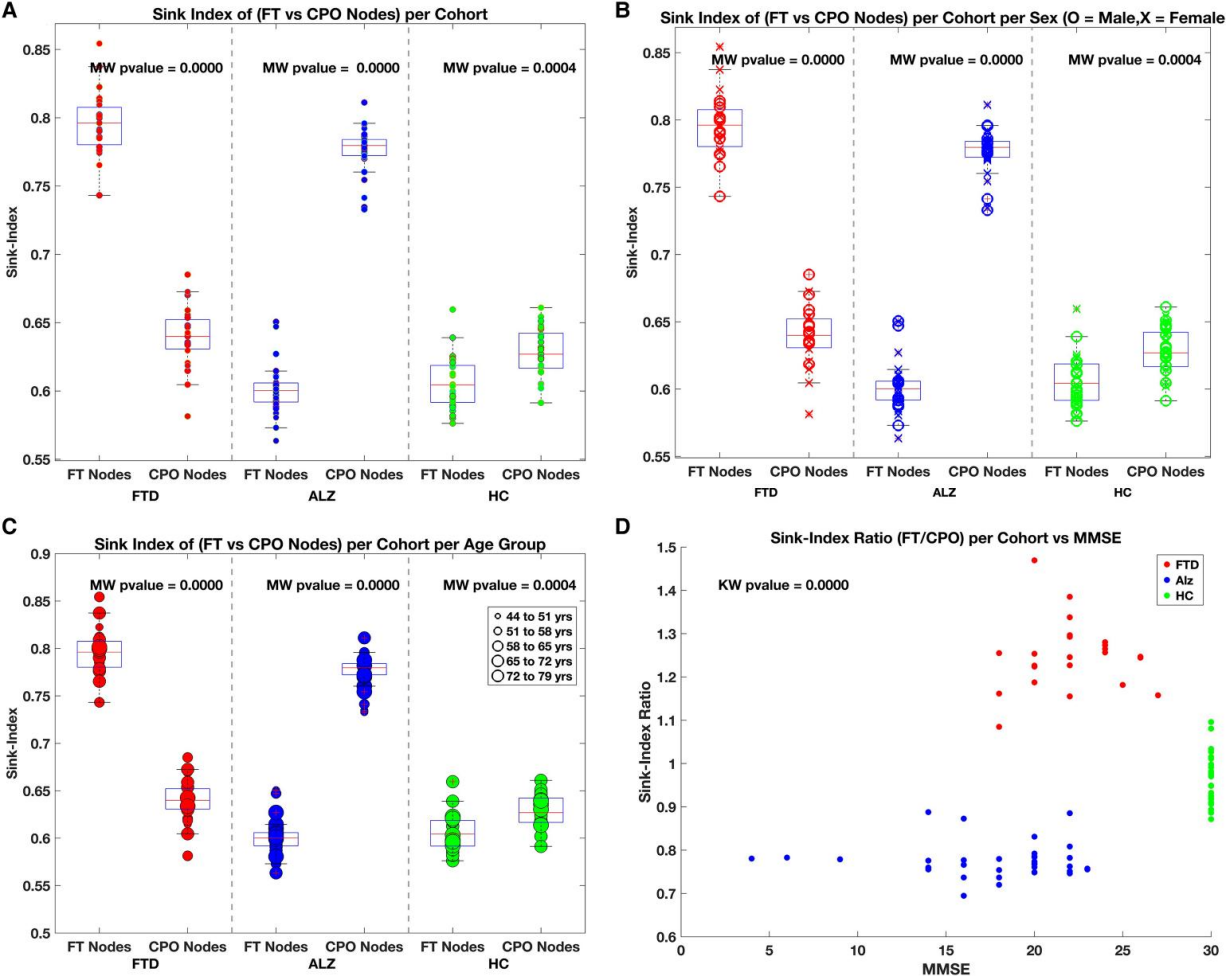
**Cohort-level Sink-Index and MMSE behaviour.** In (**A**), the average sink indices for the frontal–temporal (FT) and central-–parietal–occipital (CPO) regions for each subject of each cohort, AD (*N* = 36), FTD (*N* = 23), and HC (*N* = 29), capture the subject’s brain region dynamics. HCs display closely related sink indices between FT and CPO regions. Each individual data point in Panels A–C represents the average Sink-Index for a single subject in either the FT or CPO node group while each point in Panel D represents the Sink-Index ratio (FT/CPO) of a single subject plotted against their MMSE score. Population characteristics such as Sex (**B**) and Age (**C**) do not seem to bias the average sink indices. In contrast, the MMSE score as a function of the Sink-Index ratio (**D**) shows similar scores corresponding to highly dispersed (high and low) sink indices across both the AD and FTD groups. For Panels A–C, the Mann–Whitney U-test yielded significant differences between the FT and CPO nodes (*P* = 0.0000) for the AD and FTD groups; similarly for the nodes of the HC group (*P* = 0.0004). In Panel D, the Kruskal–Wallis statistical test identified significant differences (*P* < 0.001) across groups (FTD, HC, and Alz).

Panel A of Fig. 5 summarizes our population-level findings following the approach used to analyse subjects 1–3 from Fig. 3. For each group, we captured the average Sink-Index for the entire recording for all channels in the FT versus the CPO regions. We observed that the Sink-Index of the EEG electrodes associated to the FT brain regions (Fp1, Fp2, F3, F4, F7, F8, T3, T4) and CPO regions (O1, T5, O2, T6, Cz, Pz, P3, Fz, P4, C3, and C4) display noticeable differences between FTD (1.3389 ± 0.0895 versus 0.8444 ± 0.0651), ALZ (0.6015 ± 0.0188 versus 0.7766 ± 0.0158) and HC (0.8978 ± 0.0453 versus 0.9116 ± 0.0457). Several studies have reported differences in FTD and AD subjects, depending on sex and age, suggesting implications for pathogenesis and clinical features in the pathophysiology.^36,37^

Panel B depicts the Sink-Index per cohort and sex (female or male), while Panel C captures the Sink-Index behaviour for all three groups by age. We used five age groups, namely, 44–51 (group 1), 52–58 (group 2), 59–65 (group 3), 66–72 (group 4), and 73–79 (group 5) years old. Early-onset AD (EOAD) subjects are typically younger than 65 years old at the beginning of the illness.^38^ The data set contains 16 subjects who were younger than 65 years at the time of EEG recording. The Sink-Index identified those subjects in age groups 1 and 1–3 for AD. Table 2 lists the early-onset participants and their demographics. Bonferroni corrected *P*-values from Mann–Whitney U-tests revealed that FT nodes display statistically highly significant (*P* < 0.001, U = 0) sink indices compared to the CPO nodes, regardless of gender and age. Finally, Panel D shows a plot of the average Sink-Index against MMSE score for each subject. Several subjects from the FTD and AD cohorts had equal MMSE but different Sink-Index ratios (Sink-Index of FT regions divided by Sink-Index of CPO regions). Similarly, the HC group all had MMSE scores of 30 with a Sink-Index ratio spreading between 0.85 and 1.1. The Kruskal–Wallis test identified highly significant differences (*P* = 0.0000, *H* = 76) for the Sink-Index Ratio across groups (FTD, HC, and Alz).

**Table 2:** Early-onset demographics

Participant	Gender	Age	Condition	MMSE	Age Group
sub-028	M	49	A	20	1
sub-029	F	53	A	16	2
sub-030	F	56	A	20	2
sub-001	F	57	A	16	2
sub-035	F	57	A	22	2
sub-036	F	58	A	9	2
sub-032	F	59	A	20	3
sub-023	M	60	A	16	3
sub-006	F	61	A	14	3
sub-015	M	61	A	18	3
sub-017	F	61	A	6	3
sub-026	F	61	A	18	3
sub-008	M	62	A	16	3
sub-019	F	62	A	14	3
sub-012	M	63	A	18	3
sub-013	F	64	A	20	3

Early onset (EO) for Alzheimer < 65. Dataset contains 16 EO participants in AD cohort.

### The sink-Index ratio is a robust classifier

The Sink-Index captures the dynamics occurring among the channels in the FT Regions and those in the CPO regions. To create a single classification feature, we divided the FT regions’ Sink-Index by the CPO regions’ Sink-Index. We applied the same grouping and ratio concepts to the features provided by each analysis technique to obtain a single classifier. Namely, for the frequency analysis, we used the power ratio for FT and CPO groups, and for the Hjorth Parameters, we used PCA-reduced Hjorth parameters, five parameters total.

**Figure 6:**
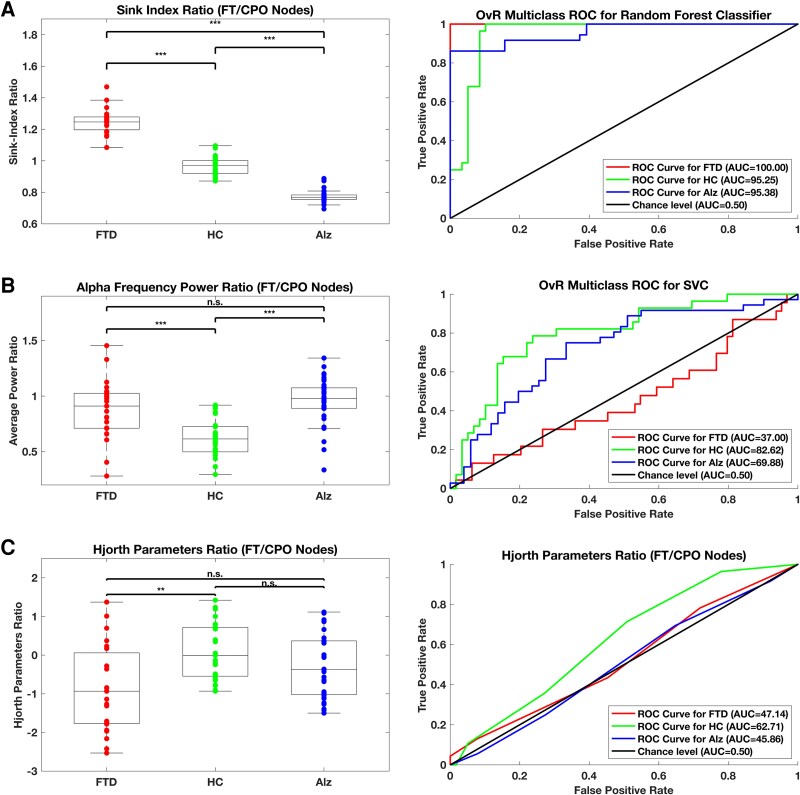
**Boxplot and ROC for classifier feature.** We used box plots to visually indicate the distribution of each feature result across the 25th, 50th, and 75th percentiles and to locate outliers in the left panels, applying the ratio between frontal–temporal and central–parietal–occipital regions for each feature. We plot the best classifier algorithm ROC corresponding to each feature in the right panels. Panels (**A**), (**B**), and (**C**) reflect Sink-Index Ratio, Alpha Frequency Power Ratio, and Hjorth Parameters Ratio respectively for 88 subjects in total: AD (*N* = 36), FTD (*N* = 23), and HC (*N* = 29). Each individual data point in Panels A–C represents the FT/CPO ratio calculated from a single subject’s EEG data using the respective metric. In all panels, the Kruskal–Wallis statistical test identified significant differences (*P* < 0.01) across groups (FTD, HC, and Alz). *Post-hoc* Dunn’s test was subsequently applied for pairwise comparisons to identify group-specific differences. In Panel A, the FTD group had significantly higher Sink-Index Ratios than both HC (*P* = 0.0008) and Alz (*P* < 0.0001). Additionally, HC exhibited higher Sink-Index Ratios than Alz (*P* < 0.0001). In Panel B, the FTD group showed significantly higher Alpha Frequency Power Ratios compared to HC (*P* = 0.0007) but not compared to Alz (*P* = 0.5699). HC exhibited significantly lower Alpha Frequency Power Ratios than Alz (*P* < 0.0001). In Panel C, the FTD group showed significantly lower Hjorth Parameters Ratios compared to HC (*P* = 0.0015), while no significant differences were found between HC and Alz (*P* = 0.1550) or between FTD and Alz (*P* = 0.2050).

Figure 6 depicts the feature distributions using box plots (left panels) and the best-performing multiclass ROCs (right panels) using the One-versus-the-Rest (OvR) multiclass approach. In Panel 6A, the Sink-Index ratio boxplot displays interquartile distributions for the FTD, HC, and AD groups with median Sink-Index ratio values of 1.24, 0.97, and.76, respectively. The boxplot summaries for each cohort show a minimum to zero IQR overlap. The corresponding Sink-Index ratio ROCs from a Random Forest Classifier display AUCs of.96, 0.99, and.95 for AD, FTD, and HC. In Panel 6B, the IQR for the HC cohort shows some overlap with FTD and AD for the Alpha band frequency power feature. However, between pathologies, IQR overlap was evident. The corresponding frequency power ROCs from a Support Vector Classifier (SVC) display AUCs of.69, 0.38, and.79 for AD, FTD, and HC. Conversely, in Panel 6C, the Hjorth parameter feature displays total IQR overlap across all cohorts. The corresponding Hjorth parameter ROCs display AUCs barely above or at chance levels of.50, 0.53, and.62 for AD, FTD, and HC.

In Fig. 6, the Kruskal–Wallis test identified very significant differences (*P* < 0.01) across groups (FTD, HC, and Alz) for the Sink-Index Ratio (Panel A), Alpha Frequency Power Ratio (Panel B), and Hjorth Parameters Ratio (Panel C). In Panel A, Dunn’s pairwise comparisons revealed that the FTD group had highly significant Sink-Index Ratios compared to HC (*P* = 0.0008, z = 2.1082) and Alz (*P* = 0.0000, *z* = 4.1466), while HC also showed higher Sink-Index Ratios compared to Alz (*P* = 0.0000, *z* = 7.0047). Panel B results showed that the FTD group had higher Alpha Frequency Power Ratios compared to HC (*P* = 0.0007, *z* = 3.0074) but not compared to Alz (*P* = 0.5699, z = 1.0731). However, HC exhibited highly significant lower Alpha Frequency Power Ratios compared to Alz (*P* = 0.0000, *z* = 4.5133). In Panel C, the Hjorth Parameters Ratio was lower in the FTD group compared to HC (*P* = 0.0015, *z* = 2.8567), while no statistically significant differences were detected between HC and Alz (*P* = 0.1550, *z* = 1.5930) or between FTD and Alz (*P* = 0.2050, *z* = 1.4924). These findings highlight the ability of the Sink-Index Ratio to distinguish between the three groups, the Alpha Frequency Power Ratio to differentiate HC from both FTD and Alz, but not between FTD and Alz, and the Hjorth Parameters Ratio to separate FTD from HC but not from Alz.

### Sink-Index ratio outperforms features from other analysis techniques

**Figure 7:**
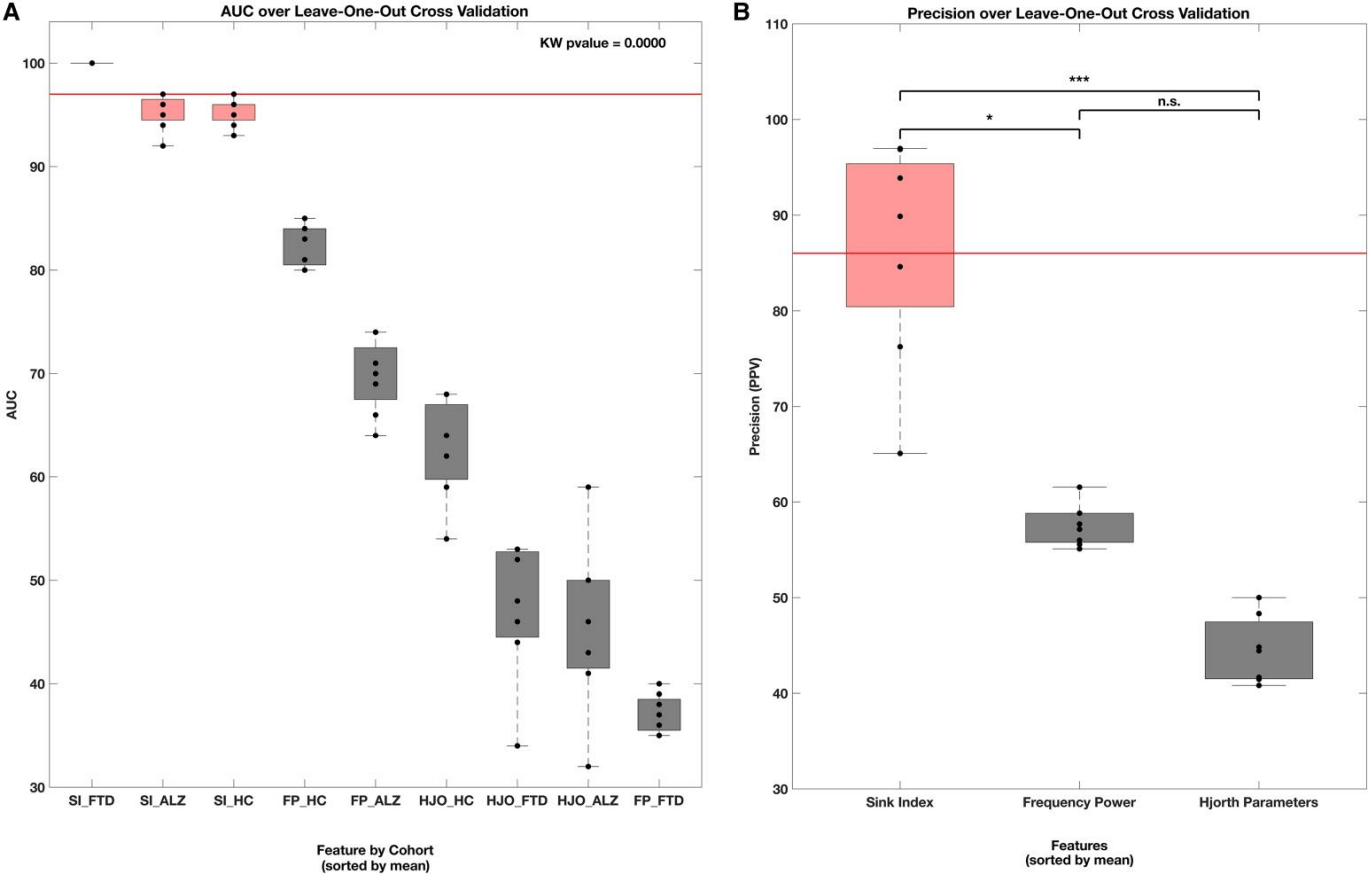
**Average AUC and precision performance benchmarks.** Panel (**A**) shows the Area Under the Curve (AUC) and Panel (**B**) displays the Precision (Positive Predictive Value, PPV), both obtained from repeated Leave-One-Out Cross Validation (LOOCV) for each feature type and cohort, sorted by mean. Each box represents the distribution of scores across 8 independent LOOCV repetitions, each performed over the full dataset of 88 subjects (FTD: *N* = 23; Alzheimer’s: *N* = 36; Healthy Controls: *N* = 29). The Sink-Index outperformed all other features in both AUC and Precision metrics. The average AUC for Sink-Index across all groups was 96.87 ± 2.54, while the second-best feature, Frequency Power, showed 63.16 ± 19.78. For Precision, Sink-Index achieved 84.39 ± 16.54, significantly outperforming Frequency Power, which yielded 57.58 ± 2.12. Hjorth Parameters showed the lowest performance in both metrics. A Kruskal–Wallis test revealed a highly significant difference across groups (*P* < 0.0001). *Post-hoc* Dunn’s tests (Bonferroni-adjusted) identified significant pairwise differences between SI_FTD and FP_ALZ (*P* = 0.0406), HJO_HC (*P* = 0.0061), HJO_FTD (*P* = 0.0029), HJO_ALZ (*P* = 0.0024), and FP_FTD (*P* = 0.0003), but no significant differences between SI_FTD and SI_ALZ (*P* = 1.0000), or between SI_ALZ and SI_HC (*P* = 1.0000). Panel (**B**) shows the aggregated precision per feature across all subjects (*N* = 88). The Kruskal–Wallis test again revealed a significant difference across features (*P* = 0.0000), with the Sink-Index significantly outperforming Hjorth Parameters (*P* = 0.0000). The difference between Sink-Index and Frequency Power approached significance (*P* = 0.0548), while Frequency Power and Hjorth Parameters did not differ significantly (*P* = 0.0976).

Random Forest performs best for the Sink-Index ratio compared to all other classification techniques. For the power spectrum ratio, in Fig. 6 Panel B, SVC performs the best compared to all other classification techniques. However, the power spectrum ratio ROC for the Alpha frequency shows diminished performance. Figure 7 depicts the average AUC and precision performance benchmarks per group, sorted by mean for each analysis technique. The AUC for Sink-Index across all groups presented values of 96.87 ± 2.54, while the power spectrum ratio and Hjorth parameters resulted in 63.16 ± 19.78 and 51.90 ± 10.22, respectively. Panel A shows how the AUC of the Sink-Index is more than 12% higher for the Sink-Index ratio feature in HCs 95.25 ± 1.28 versus the subsequent best representation, the power spectrum ratio 82.62 ± 1.99.

For Fig. 7 Panel A, a Kruskal–Wallis test showed a highly significant result (*P* = 0. 0000, *H* = 65.1157), indicating that the medians of the distributions of features differed between at least one pair of groups. The Dunn's test results, with Bonferroni-adjusted *P*-values, highlighted several pairwise differences, particularly involving Group 1 (SI_FTD). For instance, Group 1 differed significantly from Group 5 (FP_ALZ) with (*P* = 0.0406, *z* = 1.9966), from Group 6 (HJO_HC) with (*P* = 0.0061, z = 2.3058), and from Groups 7, 8, and 9 (HJO_FTD, HJO_ALZ, FP_FTD) with (*P* = 0.00) and (z = 2.9816, 3.0533 and 3.6108 respectively). Additionally, Groups 2 (SI_ALZ) and 3 (SI_HC) also exhibited significant differences when compared to Groups 7, 8, and 9 (*P* < 0.01, 2.2425 < z < 2.9070). In contrast, no significant differences were observed between Groups 1 (SI_FTD) and 2 (SI_ALZ) (*P* = 1.000, z = 0.7030) or between Groups 2 (SI_ALZ) and 3 (SI_HC) (*P* = 1.000, z = 0.0612). These findings indicate that while the Sink-Index (SI) AUC of FTD, Alzheimer’s, and Healthy Control cohorts display some overlap, the Frequency Power (FP) and Hjorth Parameters (HJO) features yielded distinct AUCs, particularly within the pathological cohorts.

Precision measures how well the model can identify the positive class. We determine it by dividing the total correct positive predictions (true positives) by the sum of all predictions classified as positive, including both correct (true positives) and incorrect (false positives) predictions. In our multiclass classification problem, the positive class is one, and the negative class is the composition of the remaining two. Figure 7 Panel B shows precision per feature sorted by mean. The average Sink-Index Precision of 86.02 ± 11.03 outperforms the next best feature, frequency power 57.58 ± 2.12, by at least 27%. Statistical analysis using the Kruskal–Wallis test identified differences in medians (*P* = 0.0000, H = 19.5652), prompting pairwise comparisons. Dunn's tests with Bonferroni correction show that the Sink-Index achieves significant precision compared to Hjorth Parameters (*P* = 0.0000, z = 3.6170). The comparison between Sink-Index and Frequency Power did not have statistical significance (*P* = 0.0548, z = 1.9324). Frequency Power and Hjorth Parameters demonstrate no statistically significant differences in precision (*P* = 0.0976, *z* = 1.7502).

## Discussion

Recent advances in fluid biomarker research for FTD have increased understanding of its pathology, highlighted neurofilament light chain as a key marker, and opened pathways for multimodal biomarker studies to support diagnosis, prognosis, and therapeutic monitoring.^39^ Similarly, extensive biomarker research in Alzheimer's disease has yielded significant advancements, particularly in cerebrospinal fluid^40^ and blood markers,^41^ but challenges remain in developing tools that reliably differentiate AD from other neurodegenerative conditions like FTD.^42^ This study introduces a novel EEG-based marker, the Sink-Index, derived from subject-specific DNM, providing a compelling demonstration of the potential utility of EEG data for the differential diagnosis of FTD and AD.

The development of the Sink-Index opens new possibilities for the utilization of EEG data in the differential diagnosis of dementia. It shows promise in distinguishing between FTD and AD cases, as well as between neurodegenerative disease cases and controls, with strong classification properties relative to other EEG analysis approaches. Importantly, it provides a cortical function profile comparable to those from FDG-PET^43^ that are used for FTD and AD diagnosis. While further validation work is necessary, the potential of the Sink-Index for diagnostic-quality brain function mapping using inexpensive, widely available, portable technology is substantial. Its capability for remote measurement and monitoring in both clinical practice and research, as well as its potential for use in resource-limited environments, holds promise for longitudinal assessments of brain function in observational studies and clinical trials.

### Sink-Index as an EEG marker for FTD diagnosis

The Sink-Index highlighted regions of brain dysfunction in FTD^44^ and AD^45^ subjects, aligning with the established neurodegenerative patterns and radiological profiles of these diseases.^8^ For AD, a lower Sink-Index in FT nodes compared to CPO nodes was observed, reflecting the synaptic dysfunction and neuronal loss in regions critical for attention, mental acuity, and vigilance. Conversely, a higher Sink-Index in FT nodes in FTD highlighted the predilection for neuronal dysfunction and loss in areas responsible for social behaviour and language. These patterns are likely influenced by structural degeneration, as atrophy reduces synaptic density and connectivity in specific regions.^25^ For FTD, the higher Sink-Index in the FT regions corresponds well with the characteristic focal atrophy seen in the frontal and anterior temporal lobes, disrupting outgoing connectivity and resulting in these regions acting predominantly as ‘sinks’ in the network. In contrast, for AD, the Sink-Index has relatively higher values in CPO regions, aligning with diffuse atrophy with a predilection for the hippocampus, medial temporal lobe, and temporoparietal cortex.^26^ These findings are consistent with neuroimaging studies that report focal atrophy in FTD and diffuse patterns of atrophy in AD, emphasizing the translational relevance of the Sink-Index as a marker that reflects both functional and structural changes.

The Sink-Index Ratio emerged as a robust classifier, distinguishing between AD, FTD, and HCs with high accuracy, as evidenced by the high AUC values in Fig. 7 Panel A. This novel metric offers diagnostic potential and may provide insights into compensatory mechanisms in the brain. For instance, in AD, the altered network dynamics may reflect regional loss of functions, as well as neurophysiological adaptations of the network to maintain functional connectivity. With respect to FTD, the increase in the Sink-Index in FT nodes most likely reflects focal neuronal dysfunction and loss. These interpretations require further validation. The implications of our findings extend beyond diagnostics. While MRI assesses atrophy patterns and FDG-PET captures distributions of metabolic activity,^46,47^ the Sink-Index, derived from subject-specific DNM from a Scalp EEG, captures alterations in brain functional connectivity. This offers a potential adjunct to standard brain imaging for observational studies and clinical trials. By capturing dynamic interactions in neural networks, the Sink-Index provides a unique perspective on the intricate neural and network interactions vital for understanding how neurodegenerative diseases manifest as clinical syndromes.

### Comparisons to previous studies in resting-state EEG markers for dementia

Resting-state EEG (rsEEG) has been previously explored for its potential to differentiate between Alzheimer's disease (AD) and frontotemporal dementia (FTD). Earlier studies have demonstrated that rsEEG can capture disease-specific patterns, such as reduced signal complexity in AD using measures like ApEn^16-17^ and SampEn,^18^ and regional hypoconnectivity in behavioural variant frontotemporal dementia (bvFTD) compared to AD.^48^ Spectral analyses have highlighted distinctive rhythm abnormalities, with slowing in delta and theta bands observed in AD and preserved alpha activity in FTD.^49^ Time-frequency analyses using techniques like Wavelet Transform have similarly contributed to distinguishing dementia subtypes, albeit with modest accuracy rates.^24^ However, most studies are limited by small sample sizes, focus on comparisons of disease groups with healthy controls (e.g. AD versus HC or FTD versus HC), or use methods that fail to account for dynamic, network-wide interactions in the brain.

This study addresses these limitations by analysing a larger dataset of 88 subjects and examining disease group comparisons. Utilizing a time-domain approach to extract the Sink-Index ratio, it overcomes the need for predefined frequency bands and captures n-to-n regional dynamics across the brain network. The DNMs derived from subject-specific EEG data provide a richer characterization of natural frequencies and channel influences, offering a more comprehensive framework for biomarker development. Despite these advancements, challenges remain in clinically validating rsEEG biomarkers for dementia. Standardized protocols, larger datasets, and integration with multimodal data are necessary to translate these findings into reliable diagnostic tools. The early and accurate differentiation of AD and FTD is critical for enabling timely intervention and improving patient care, emphasizing the need for continued innovation in this field.

### Study limitations

EEG channels capture electrical activity from different brain regions, which, through synchronous activation, reflect the activity of functional networks. In this sense, EEG provides an alternative method to infer brain network dynamics, similar to modalities like MRI or MEG. The activity captured by the EEG channels mirrors the local activity of the underlying brain regions. The frontal–temporal (FT) and central–parietal–occipital (CPO) distributions of electrodes in this study allow for a reasonable approximation of the functional connectivity networks relevant to AD and FTD. However, our approach is based on approximations, as our electrode distributions do not replicate the nodes published in functional or structural network studies of FTD and AD. We acknowledge this limitation and propose that future prospective studies optimize electrode number and placement to improve the anatomical and functional specificity of EEG-based network analyses. Additionally, incorporating standard atlases, such as the Desikan–Killiany atlas, could help refine the localization of brain regions and facilitate stronger comparisons with established network definitions. These refinements would help validate and enhance the robustness of the Sink-Index as a biomarker for neurodegenerative diseases.

Furthermore, the size of our sample is limited, and though it was sufficient for the preliminary descriptions reported here, we acknowledge the need for validation in larger-scale studies. Also, the cases in this study did not undergo pathological confirmation of the diagnosis (which is typically achieved by post-mortem brain examination or by genetic analysis in familial cases). We would also note that the complexity of EEG signals and the individual variability observed in this study, particularly in the AD cases, underlines the need for refinement of the Sink-Index metric to optimize clinical utility.

We also acknowledge the limitation of reliance on Mini-Mental State Examination (MMSE) scores, a bedside measure of global cognitive impairment that does not capture the types of behavioural impairments typical of frontotemporal dementia (FTD). Furthermore, there were differences in MMSE scores between the AD and FTD groups (mean: 17.75 ± 4.5 versus 22.1 ± 2.6), which complicate direct comparisons. While the MMSE was chosen for its availability and widespread use as a standard measure, future studies should incorporate more tailored neuropsychological assessments and recruit cohorts with comparable MMSE scores. This approach would help disentangle the effects of disease-specific network dysfunction from global cognitive decline, providing a deeper understanding of the Sink-Index’s diagnostic utility.

### Future directions

While this study demonstrates the potential of the Sink-Index (SI) as a robust EEG marker for distinguishing neurodegenerative conditions, several key areas warrant further exploration to enhance its applicability and generalizability.

### Validation with larger cohorts

The relatively small cohort size (*n* = 88) in this study, drawn from publicly available EEG datasets, represents an inherent limitation. Although the use of leave-one-out cross-validation (LOOCV) minimized bias and ensured reliable classification performance, larger, independent datasets are needed to confirm these findings. Future work will prioritize the inclusion of multi-center datasets to validate the Sink-Index across diverse populations and clinical settings. This effort will not only improve the robustness of the Sink-Index but also establish its utility in broader diagnostic frameworks.

### Lateralization patterns in neurodegenerative diseases

Lateralization of brain network dynamics is a hallmark of certain neurodegenerative diseases, particularly FTD, which is often characterized by left-sided frontal–temporal (FT) atrophy. While this study primarily focused on overall Sink-Index values, preliminary observations suggest asymmetry in FT regions for FTD and more bilaterally distributed patterns in central–parietal–occipital (CPO) regions for AD. Future research will systematically quantify these hemispheric differences, examining their relationship with clinical features such as language and motor deficits.^50^ A deeper understanding of lateralization patterns could refine the Sink-Index’s diagnostic precision and provide insights into disease-specific network dynamics.

### Application to FTD categories defined by neuropathological classification

Expanding the Sink-Index’s application to tauopathies, for example, such as corticobasal degeneration (CBD) and progressive supranuclear palsy (PSP), represents an exciting direction for future research. These conditions exhibit distinct neurodegenerative patterns, with CBD characterized by asymmetric motor-parietal degeneration and PSP involving midbrain atrophy and frontal dysfunction.^51^ The Sink-Index may show value of diagnostic classifications and may, with further development, provide valuable insights regarding network disruptions, pertaining to motor-parietal regions for CBD and frontal-midbrain dynamics for PSP.

## Conclusion

In conclusion, our research introduces a novel EEG marker for FTD and AD. Through its portrayal of neural network dynamics, the Sink-Index makes possible the use of scalp EEG as a non-invasive, cost-effective diagnostic tool. The effectiveness of the Sink-Index in identifying the characteristic neurodegenerative patterns of FTD and AD suggests high potential for transforming aspects of the diagnostic process for these conditions. Future studies, primarily involving larger cohorts and longitudinal data, are necessary to validate and refine this tool, with the expectation that this will ultimately lead to its integration into clinical practice and research.

## Data Availability

The data used in this study come from a publicly available dataset located at OpenNeuro and accessible via the following link: https://openneuro.org/datasets/ds004504/versions/1.0.4
